# Tracking platyhelminth parasite diversity from freshwater turtles in French Guiana: First report of *Neopolystoma* Price, 1939 (Monogenea: Polystomatidae) with the description of three new species

**DOI:** 10.1186/s13071-017-1986-y

**Published:** 2017-01-31

**Authors:** Louis H. Du Preez, Mathieu Badets, Laurent Héritier, Olivier Verneau

**Affiliations:** 1 Unit for Environmental Sciences and Management, North-West University, Potchefstroom, 2520 South Africa; 2 South African Institute for Aquatic Biodiversity, Somerset Street, Grahamstown, 6139 South Africa; 3 University of Perpignan Via Domitia, Centre de Formation et de Recherche sur les Environnements Méditerranéens, UMR 5110, F-66860 Perpignan, France; 4 CNRS, Centre de Formation et de Recherche sur les Environnements Méditerranéens, UMR 5110, F-66860 Perpignan, France

**Keywords:** French Guiana, Freshwater turtle, *Kinosternon scorpioides*, *Mesoclemmys gibba*, *Rhinoclemmys punctularia*, Polystomatidae, *Neopolystoma*

## Abstract

**Background:**

Polystomatid flatworms in chelonians are divided into three genera, i.e. *Polystomoides* Ward, 1917, *Polystomoidella* Price, 1939 and *Neopolystoma* Price, 1939, according to the number of haptoral hooks. Among the about 55 polystome species that are known to date from the 327 modern living chelonians, only four species of *Polystomoides* are currently recognised within the 45 South American freshwater turtles.

**Methods:**

During 2012, several sites in the vicinity of the cities Cayenne and Kaw in French Guiana were investigated for freshwater turtles. Turtles were collected at six sites and the presence of polystomatid flatworms was assessed from the presence of polystome eggs released by infected specimens.

**Results:**

Among the three turtle species that were collected, no polystomes were found in the gibba turtle *Mesoclemmys gibba* (Schweigger, 1812). The spot-legged turtle *Rhinoclemmys punctularia* (Daudin, 1801) was infected with two species of *Neopolystoma* Price, 1939, one in the conjunctival sacs and the other in the urinary bladder, while the scorpion mud turtle *Kinosternon scorpioides* (Linnaeus, 1766) was found to be infected with a single *Neopolystoma* species in the conjunctival sacs. These parasites could be distinguished from known species of *Neopolystoma* by a combination of morphological characteristics including body size, number and length of genital spines, shape and size of the testis. They were also differentiated at the molecular level using the *cox*1 gene marker. Based on morphological and genetic evidences, three new species are described herein, namely *Neopolystoma cayensis* n. sp. and *Neopolystoma guianensis* n. sp. from the bladder and the conjunctival sacs of *R. punctularia*, respectively, and *Neopolystoma scorpioides* n. sp. from the conjunctival sacs of *K. scorpioides*. However the monophyly of *Polystomoides* and *Neopolystoma* is still questioned regarding their phylogeny based on a dataset comprising four concatenated genes, namely, 18S, 28S and 12S rRNA genes and *cox*1.

**Conclusions:**

In addition to these being the first chelonian polystomes to be reported and described from French Guiana, they represent the first polystomes from the hosts *K. scorpioides* and *R. punctularia* and the first representatives of *Neopolystoma* from South America. Chelonian polystomes now require an in-depth morphological study to reconcile the taxonomy of the genera with species evolution.

## Background

While freshwater makes up less than 1% of the earth's surface, it supports at least 100,000 animal species, approximately 9.5% of the animal diversity [1, 2]. As a starting point for conservation of the freshwater biodiversity, a comprehensive map grounded mainly on fish taxonomic studies was developed to design world’s freshwater ecoregions [3]. Based on fish species richness and endemicity, several areas were identified as being of significant importance, including large portions of the Amazon basin, among which the Guianas ecoregion [3]. Besides its exceptional richness in freshwater fishes [3], this ecoregion also exhibits a great diversity of freshwater turtles with ten species known to date [4]. These include the matamata turtle *Chelus fimbriata* (Schneider, 1783), the scorpion mud turtle *Kinosternon scorpioides* (Linnaeus, 1766), the gibba turtle *Mesoclemmys gibba* (Schweigger, 1812), the Guyanan toad-headed turtle *Mesoclemmys nasuta* (Schweigger, 1812), the big-headed sideneck turtle *Peltocephalus dumerilianus* (Schweigger, 1812), the Guianan shield side-necked turtle *Phrynops tuberosus* (Peters, 1870), the twist-necked turtle *Platemys platycephala* (Schneider, 1792), the giant South American river turtle *Podocnemis expansa* (Schweigger, 1812), the yellow-spotted river turtle *Podocnemis unifilis* Troschel, 1848 and the spot-legged turtle *Rhinoclemmys punctularia* (Daudin, 1801).

Globally turtles are known to be very sensitive to modifications of their environment. They are often among the first vertebrates to disappear from impacted or altered habitats [5] and, as a result, they represent one of the most endangered groups of animals [6, 7]. Of the 327 recognised species of extant chelonians (turtles and tortoises) that are distributed throughout the most hospitable ecoregions [4], 63% are considered threatened [6]. The main reasons for the decline is degradation and loss of habitat, but over-exploitation for the food market [8], pet trade [9], biological invasions [10–12] and diseases [13, 14] are also contributing causes. Freshwater turtles are indeed hosts to a wide variety of pathogens and parasites representing all major parasitic groups, including viruses [15], bacteria [16], blood parasites [17] and helminths [18, 19]. Among them, the Polystomatidae (Platyhelminthes: Monogenea) comprises 25 genera of which three, i.e. *Polystomoides* Ward, 1917, *Polystomoidella* Price, 1939 and *Neopolystoma* Price, 1939 infect freshwater turtles [20]. Only about 55 polystome species are known to date within chelonians, which represents approximately 25% of all known extant polystomes. Chelonian polystomes are worldwide distributed and form a clade that might have originated 178 million years ago, following a switch from caecilians to primitive freshwater turtles [21]. They would have secondarily diversified within hosts as a consequence of plate tectonics, co-divergences, turtle dispersal followed by host switching, intra-host speciation or a mixture of all these events [21]. Regardless of the scenarios involved in host-parasite diversification, these parasites are mostly host- and site-specific [22]. However, they appear to be less specific following host releases, which has been demonstrated in confined and natural environments after translocation of American freshwater turtles, especially red-eared sliders *Trachemys scripta elegans* (Wied, 1939) [23–25].

Since the trade of sliders was banned in Europe in 1997 [9], an increasing number of disparate freshwater turtles globally appeared in the pet trade, providing a pool of new species susceptible to be introduced into natural environments and pose a high risk for European wetlands due to climate matching [9]. As the pet trade is growing, the list of traded turtles may change rapidly in response to supply and demand [9]. Hence, there is an urgent need to assess the parasite diversity of freshwater turtles in their area of origin before new species of parasites are introduced with their native hosts in novel environments. Due to the high diversity of turtles in the Guianas that could be potentially sold as pets in Europe and some other non-European countries, our first survey of platyhelminth parasites took place in this ecoregion. We first concentrated in French Guiana freshwater environments to collect and examine freshwater turtles and their platyhelminth parasites. We report and describe hereafter three yet unknown chelonian polystomes from two host species, *K. scorpioides* and *R. punctularia*.

## Methods

### Host sampling

During the period 9–23 April 2012, several swampy areas and ponds near Cayenne and Kaw in French Guiana were investigated for freshwater chelonians. Site A (4.89205N, 52.34643W), site B (4.90149N, 52.35767W), site C (4.82301N, 52.34115W), site D (4.66997N, 52.30560W) and site E (4.87901N, 52.25644W) were all water bodies in and around Cayenne, while site F (4.87082N, 52.33678W) was a forested pond on the road to the town of Kaw. Crayfish traps baited with pork or ox liver were anchored to the vegetation to ensure they did not roll into the deeper water. They were left out overnight and placed in such a way as to allow turtles to surface and breathe when trapped. Captured animals were removed from traps the following day and placed individually in plastic tubs containing water about 50 mm deep and covered with shelving to keep tubs cool and dark and to prevent turtles from escaping.

### Parasite sampling

The day after turtle’s isolation, the water was poured through two plankton sieves with respective mesh size of 500 and 100 μm. The 500 μm sieve retained course debris and most of the faeces while the 100 μm sieve retained parasite eggs. The content of the 100 μm sieve was then washed into a Petri dish and inspected for the presence of polystome eggs using a Nikon SMZ 645 dissecting microscope. Turtles for which no parasite eggs were detected were screened on a second and third day and, if no polystome eggs were detected, animals were released at the point of collection. Turtles to be dissected were killed by means of a lethal injection of 0.5 ml Uthapent (sodium pentabarbitone) diluted with 4.5 ml water. The urinary bladder and all reproductive and excretory ducts, as well as the oral and nasal cavities and conjunctival sacs under the eyelids, were examined for polystomes. All visible parasites were removed, after which the host tissue was placed in hot 70% ethanol and vigorously shaken to detach any immature parasites that might have been overlooked. Live parasites were immediately placed in a drop of water on a specimen glass slide and briefly heated from below with a butane lighter until they relaxed and stopped moving. They were fixed in 10% neutral buffered formalin under very gentle coverslip pressure and permanently mounted. Some polystome eggs were also placed in dechlorinated water in cryo vials but failed to develop. Finally, sub-adult and mature specimens earmarked for molecular studies were fixed in molecular grade 70% ethanol.

### Whole mount preparation, morphology and morphometry

Polystomes were washed free of fixative and stained overnight in a weak solution of acetocarmine, dehydrated, cleared in xylene and mounted in Canada balsam. Specimens were examined using a Nikon Eclipse E800 compound microscope as well as a Nikon AZ100 (Nikon, Netherlands). Body and organs were measured using the Nikon NIS elements software program as well as marginal hooklets that were located in mature and immature parasites. All measurements are given in micrometres as the range followed by the mean in parentheses. Drawings, based on photographs taken of type-material, were prepared using Adobe Illustrator software.

### Molecular methods

Polystome specimens were dried, crushed and incubated at 55 °C for one hour in 150 μl of 10% suspension Chelex 100 sodium (Sigma-Aldrich, L’Isle d’Abeau Chesnes, France) and proteinase K at a final concentration of 1 mg/ml. The enzymatic reaction was stopped at 100 °C for 15 min and DNA was kept at 4 °C until PCR amplification. Four molecular markers were investigated for genetic analyses: (i) the complete 18S rRNA gene was amplified in two overlapping fragments of about 1 kb each, with the primers forward F18 (5'-ACC TGG TTG ATC CTG CCA GTA G-3') and reverse 18RG (5'-CTC TCT TAA CCA TTA CTT CGG-3') on the one hand and with the primers forward 18 F3 (5'-GGA CGG CAT GTT TAC TTT GA-3') and reverse IR5 (5'-TAC GGA AAC CTT GTT ACG AC-3') on the other; (ii) partial nuclear 28S rRNA gene was also amplified in two overlapping fragments of about 1 kb and 500 bp each, with the primers forward LSU5' (5'-TAG GTC GAC CCG CTG AAY TTA AGC A-3') and reverse IR16 (5'-ATT CAC ACC CAT TGA CTC GCG-3') on the one hand and with the primers forward IF15 (5'-GTC TGT GGC GTA GTG GTA GAC-3') and reverse LSU3' (5'-TAG AAG CTT CCT GAG GGA AAC TTC GG-3') on the other; (iii) a portion of the mitochondrial 12S rRNA gene of about 470 bp was amplified with the primers forward 12SpolF1 (5'-YVG TGM CAG CMR YCG CGG YYA-3') and reverse 12SpolR1 (5'-TAC CRT GTT ACG ACT TRH CTC-3'); (iv) a fragment of the mitochondrial cytochrome *c* oxydase subunit 1 (*cox*1) gene of about 350 bp was amplified with the primers forward L-CO1p (5'-TTT TTT GGG CAT CCT GAG GTT TAT-3') and reverse H-Cox1R (5'-AAC AAC AAA CCA AGA ATC ATG-3'). PCR amplifications were conducted following the procedure described in [21]. All PCR products were purified using the kit Wizard SV Gel and PCR Clean-Up System (Promega, Charbonnières-les-Bains, France) and sent to the company Genoscreen (Lille, France) for sequencing with the PCR primers. Chromatograms were finally examined with the software Geneious (Saint Joseph, Missouri, USA) to verify all substitutions along sequences.

### Sequence alignments and phylogenetic analyses

18S and 28S sequences were aligned regarding the secondary structure of nuclear rRNAs of chelonian polystomes and subsequent alignments defined by Héritier et al. [21]. Inversely, 12S and *cox*1 sequences were aligned using Clustal W [26] implemented in the program MEGA, version 5 [27], under default parameters. In order to explore the relationships of the chelonian polystomes collected during the present study, sequences reported in [21, 24, 28–30] (GenBank accession numbers 18S: AJ228788, AJ228792, FM992696–FM992699, KR856126–KR856139; 28S: AF382065, FM992702–FM992706, KR856145–KR856158; 12S: KR856100–KR856119; *cox*1: Z83005, Z83007, Z83009, Z83011, FR822527, FR822529–FR822531, FR822534, FR822553, FR822555, FR822570, FR822587, FR822601, FR822603, FR822604, FR828360, KR856175–KR856177), as well as the newly generated sequences reported here (see below), were included in a global phylogenetic analysis that was performed following the procedure of Héritier et al. [21]. A global alignment was first inferred with the concatenated sequences recovered from 23 fully described or undescribed species. Regions not sequenced, which concerned particularly the 18S rRNA gene and the 500 bp fragment of the 28S rRNA gene of the polystome collected from the bladder of *K. scorpioides*, were treated as missing data. Ribosomal nuclear sequences were partitioned into two discrete regions, i.e. stems and loops, while 12S and *cox*1 sequences were considered as two other distinct partitions. A doublet model was considered for the stem partition and a GTR + I + G for the loops. A GTR + I + G was also selected independently for the two last partitions following the Akaike Information Criterion (AIC) implemented in Modeltest 3.06 [31]. A Bayesian analysis was run using MrBayes 3.04b [32], with four chains running for one million generations and sampled every 100 cycles. The consensus tree was then drawn after removing the first 1,000 trees (10%) as the 'burn-in' phase to obtain the Bayesian posterior probability for each association. *Nanopolystoma tinsleyi*, which was recognised as the sister group of chelonian polystomes after phylogenetic analysis [21], was used as the outgroup (GenBank accession numbers 18S: KR856124; 28S: KR856142; 12S: KR856077; *cox*1:KR856164).

### Genetic divergences within chelonian polystomes

Kimura 2-parameters (K2P) distances were estimated from the whole *cox*1 dataset using MEGA, version 5, to assess genetic divergences among chelonian polystomes. Results were discussed in the light of the 3.4% *cox*1 genetic divergence threshold defined by Héritier et al. [23] on chelonian polystomes.

## Results

### Turtles and parasites collected

The spot-legged turtle *R. punctularia* was found at all the sites except site A. Of the total sample, 23 specimens were collected at site B, three at site C, one at site D, three at site E and 10 at site F. The scorpion mud turtle *K. scorpioides* was found at all the sites except site E. Of the total sample, two specimens were collected at site A, one each at sites B and C, 13 at site D and four at site F. Of the total sample of the gibba turtle *M. gibba*, four specimens were collected at site D and three at site E.

Among the 68 turtles that were collected, two chelonian species were found to be infected with polystomes, i.e. *R. punctularia* and *K. scorpioides*, whereas *M. gibba* was uninfected. Based on the release of polystome eggs, infected turtles were euthanized and dissected to recover all polystomes. Of the 40 *R. punctularia*, infected animals were found at sites B and F. At site B, two out of 23 turtles released round polystome eggs, while another one released round and fusiform eggs. At site F, one of the ten turtles released both round and fusiform polystome eggs while a second one released only fusiform eggs. Of the 21 *K. scorpioides*, a single turtle out of the 13 collected at site D released fusiform eggs. Based on the absence of hamuli, all parasites found in the bladder and in the conjunctival sacs of their host could be considered at that stage as belonging to *Neopolystoma*.

**Class Monogenea (van Beneden, 1858)**

**Order Polystomatidea Lebedev, 1988**

**Family Polystomatidae Gamble, 1896**

### *Neopolystoma cayensis* n. sp.

***Type-host*** **:** *Rhinoclemmys punctularia* (Daudin, 1801) (Geoemydidae Theobald, 1868).

***Type-locality*** **:** Pond on the outskirts of Cayenne, French Guiana (4.87082N, 52.33678W).

***Site in host*** **:** Urinary bladder.

***Type-material*** **:** Morphological description based on 22 sexually mature worms. The holotype (NMB P394) and nine paratypes (NMB P395–P403) were deposited in the Parasitic Worm Collection, National Museum, Aliwal Street, Bloemfontein, South Africa.

***Voucher material*** **:** Remainder of specimens were deposited in the polystome collection of the North-West University, Potchefstroom, South Africa.

***Prevalence and intensity*** **:** Site B: Prevalence 13.0%; Mean intensity 12.7. Site F: Prevalence 10.0%; One specimen infected by 176 polystomes.

***Representative DNA sequences*** **:** GenBank accession numbers: KY200986 (18S), KY200988 (28S), KY200991 (12S) and KY200994 (*cox*1).

***ZooBank registration*** **:** To comply with the regulations set out in article 8.5 of the amended 2012 version of the *International Code of Zoological Nomenclature* (ICZN) [33], details of the new species have been submitted to ZooBank. The Life Science Identifier (LSID) of the article is urn:lsid:zoobank.org:pub:B55369A7-5 F88-4A72-94 F2-E0CE354484A1. The LSID for the new name *Neopolystoma cayensis* n. sp. is urn:lsid:zoobank.org:act:A4E27E97-052E-4709-84 AD-B262A1A625D4.

***Etymology*** **:** This parasite is named after Cayenne, the capital of French Guiana, where the parasite was found.

### Description

**Fig. 1:**
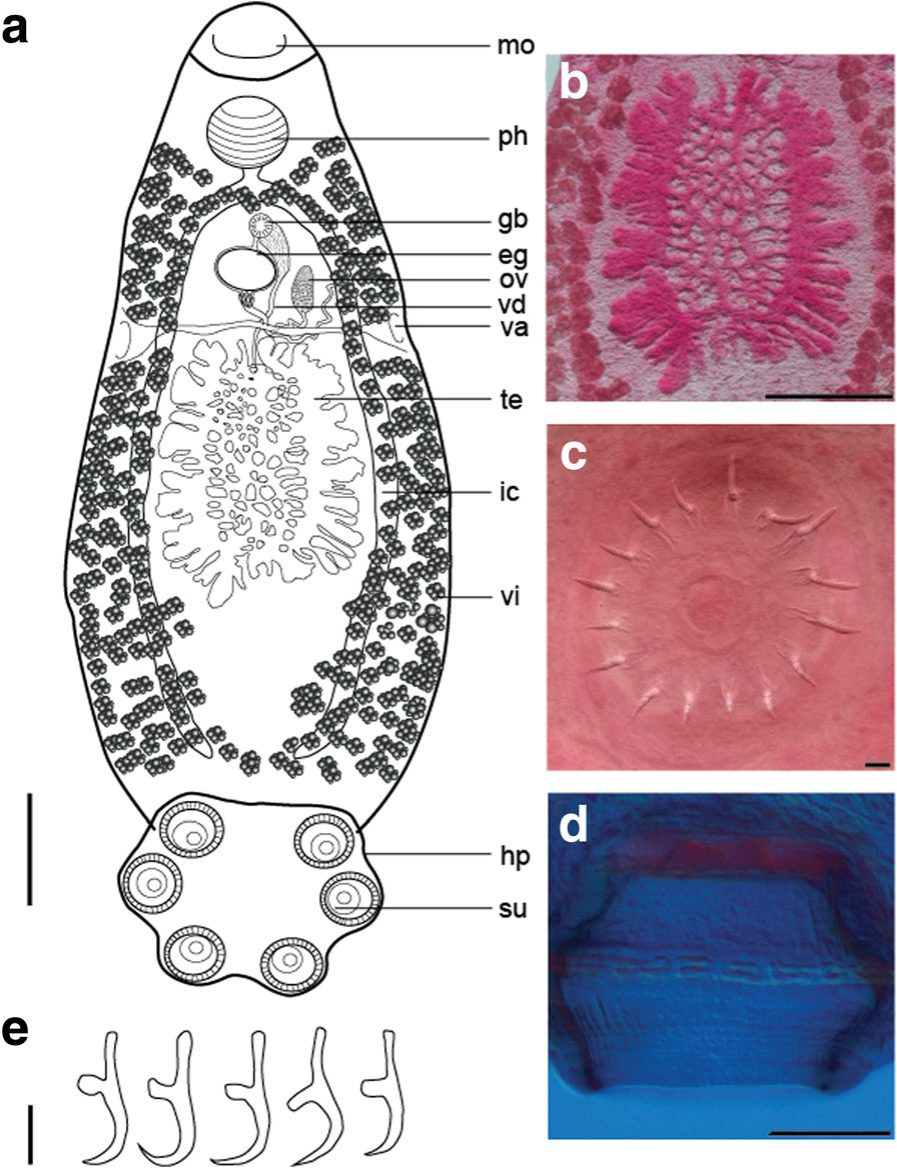
*Neopolystoma cayensis* n. sp. Holotype. **a** Ventral view. **b** Testis. **c** Genital spines. **d** Haptoral sucker showing a ring of skeletal elements. **e** Marginal hooklets. *Abbreviations*: eg, egg; gb, genital bulb; hp, haptor; ic, intestinal caecum; mo, mouth; ov, ovary; ph, pharynx; su, sucker; te, testis; va, vagina; vd, vas deferens; vi, vitellaria. *Scale-bars*: **a**, 500 μm; **b**, 500 μm; **c**, 10 μm; **d**, 100 μm; **e**, 10 μm

[Based on 22 egg-producing adults (see Table 1 and Fig. 1a-e). No larval measurements or characters are given as eggs collected from the host failed to develop.] Body elongate, pear shaped with the widest point about two-thirds from the anterior extremity (Fig. 1a), total length 3,342–5,575 (4,139), greatest width 1,353–1,961 (1,753). Haptor length 759–1,139 (912), haptor width 1,095–1,459 (1,249), haptor length to body length ratio 0.20–0.25 (0.22). Mouth subterminal, ventral. False oral sucker 130–356 (189) wide. Pharynx muscular, 293–401 × 276–457 (330 × 357). Intestine bifurcates without diverticula; caeca narrow, lacking anastomoses, run laterally, not confluent posteriorly, not extending into haptor. Testis single, mid-ventral, medial, posterior to ovary, flattened, lobed along outer margin with reticulated network of ducts in central area (Fig. 1a, b), 560–983 × 468–1,283 (762 × 933); large amounts of sperm observed in reticulated network. Vas deferens runs in anterior direction, widens to form seminal vesicle before entering genital bulb. Genital bulb median, ventral, posterior to intestinal bifurcation, 73–129 (99) in diameter, armed with single ring of 16–17 spines (Fig. 1c), 19–26 (23) long, tip of spine curved outwards. Ovary dextral, at 15% of body length from anterior extremity, small, 140–357 × 62–118 (195 × 88). Uterus short, tubular, anterior to ovary, containing single oval egg; egg capsule 209–289 × 155–187 (246 × 165). No intra-uterine development, egg operculate. Vaginae present, located about 1/3 from anterior extremity of body proper; body width at level of vagina 1,015–1,504 (1,281); body slightly indented at level of vaginae. Vitellarium in two broad lateral fields along full length of body proper except for anterior area including mouth and pharynx and posteriormost section of the body (Fig. 1a). Vitelline follicles in two lateral fields, joining in narrow strip between genital bulb and pharynx and posteriad, at level of caeca tips. Genito-intestinal canal prominent, dextral, joining intestinal caecum at level of ovary (Fig. 1a). Haptoral suckers 6, muscular, with well-developed skeletal structure inside (Fig. 1d), mean diameter 227–297 (261). Hamuli absent. Marginal hooklets (Fig. 1e) retained in mature parasites. Posteriormost marginal hooklet 1 19.6–21.8 (21.2) long; hooklets 2–8 19.4–22.3 (20.9) long.

**Table 1:** Body morphometrics for the known species of *Neopolystoma* from the Neotropical realm

	*N. cayensis* n. sp.	*N. guianensis* n. sp.	*N. scorpioides* n. sp.	*N. domitilae*	*N. fentoni*
Body length	3,342–5,575 (4,139)	2,284–3,342 (2,737)	1,512–1,770 (1,658)	4,039–4,057	1,500–2,450 (1,985)
Body maximum width	1,353–1,961 (1,753)	830–1,164 (1,037)	868–907 (894)	1,320–1,722	426–760 (568)
Haptor length	759–1,139 (912)	634–852 (755)	445–511 (485)	1,046–1,067	449–690 (571)
Haptor width	1,095–1,459 (1,249)	784–1,014 (910)	719–765 (735)	1,416–1,851	550–850 (683)
Width at vagina	1,015–1,504 (1,281)	792–1,091 (989)	837–876 (863)	–	–
Oral sucker width	130–356 (189)	189–391 (290)	240–245 (242)	370–450 (547)	240–496 (370)
Pharynx length	293–401 (330)	189–260 (213)	170–184 (177)	305–322	156–257 (216)
Pharynx width	276–457 (357)	226–283 (252)	226–231 (228)	328	185–367 (278)
Ovary length	140–357 (195)	149–243 (197)	136–187 (153)	322–402	80–245 (103)
Ovary width	62–118 (88)	71–120 (97)	66–71 (69)	112–127	55–169 (105)
Testis length	560–983 (762)	364–541 (437)	120–142 (127)	450–664	98–367 (225)
Testis width	468–1,283 (933)	380–527 (477)	246–301 (271)	289–338	78–251 (181)
Genital bulb width	73–129 (99)	49	45	241–273	30–83 (60)
Number of genital spines	16–17	8	8	20–21	8
Genital spine length	19–26 (23)	8.4–9.8 (8.9)	5.5–8.7 (7.7)	55–71	11
Number of eggs *in utero*	1	1	1	1	1
Egg length	209–289 (246)	280–344 (317)	253–279 (265)	305	245–332 (286)
Egg width	155–187 (165)	127–163 (148)	133–156 (143)	177	122–146 (136)
Haptoral sucker width	227–297 (261)	208–242 (226)	133–279 (220)	305–483	210–326 (265)
Haptor length: Body length ratio	0.22	0.28	0.29	0.26	0.29
Marginal hooklet 1 length	19.6–21.8 (21.2)	13.3–15.1 (14.3)	16.4–17.2 (16.8)	24.6	11.8–12.7 (12.5)

### *Neopolystoma guianensis* n. sp.

***Type-host*** **:** *Rhinoclemmys punctularia* (Daudin, 1801) (Geoemydidae Theobald, 1868).

***Type-locality*** **:** Pond on the outskirts of the capital Cayenne, French Guiana (4.87082N, 52.33678W).

***Site in host*** **:** Conjunctival sacs of the eye.

***Type-material***: Eleven sexually mature worms. The holotype (NMB P404) and two paratypes (NMB P405–P406) were deposited in the Parasitic Worm Collection, National Museum, Aliwal Street, Bloemfontein, South Africa.

***Voucher material:*** Remainder of specimens were deposited in the polystome collection of the North-West University, Potchefstroom, South Africa.

***Prevalence and intensity*** **:** Site B: Prevalence 4.3%; One specimen infected by two polystomes. Site F: Prevalence 20.0%; Mean intensity 11.0.

***Representative DNA sequences*** **:** GenBank accession numbers: KY200987 (18S), KY200989 (28S), KY200992 (12S) and KY200995 (*cox*1).

***ZooBank registration*** **:** To comply with the regulations set out in article 8.5 of the amended 2012 version of the *International Code of Zoological Nomenclature* (ICZN) [33], details of the new species have been submitted to ZooBank. The Life Science Identifier (LSID) of the article is urn:lsid:zoobank.org:pub:B55369A7-5 F88-4A72-94 F2-E0CE354484A1. The LSID for the new name *Neopolystoma guianensis* n. sp. is urn:lsid:zoobank.org:act:1BDAA38D-D657-4DCC-9539-F5BDEFB463B8.

***Etymology*** **:** This parasite is named after French Guiana.

### Description

**Fig. 2:**
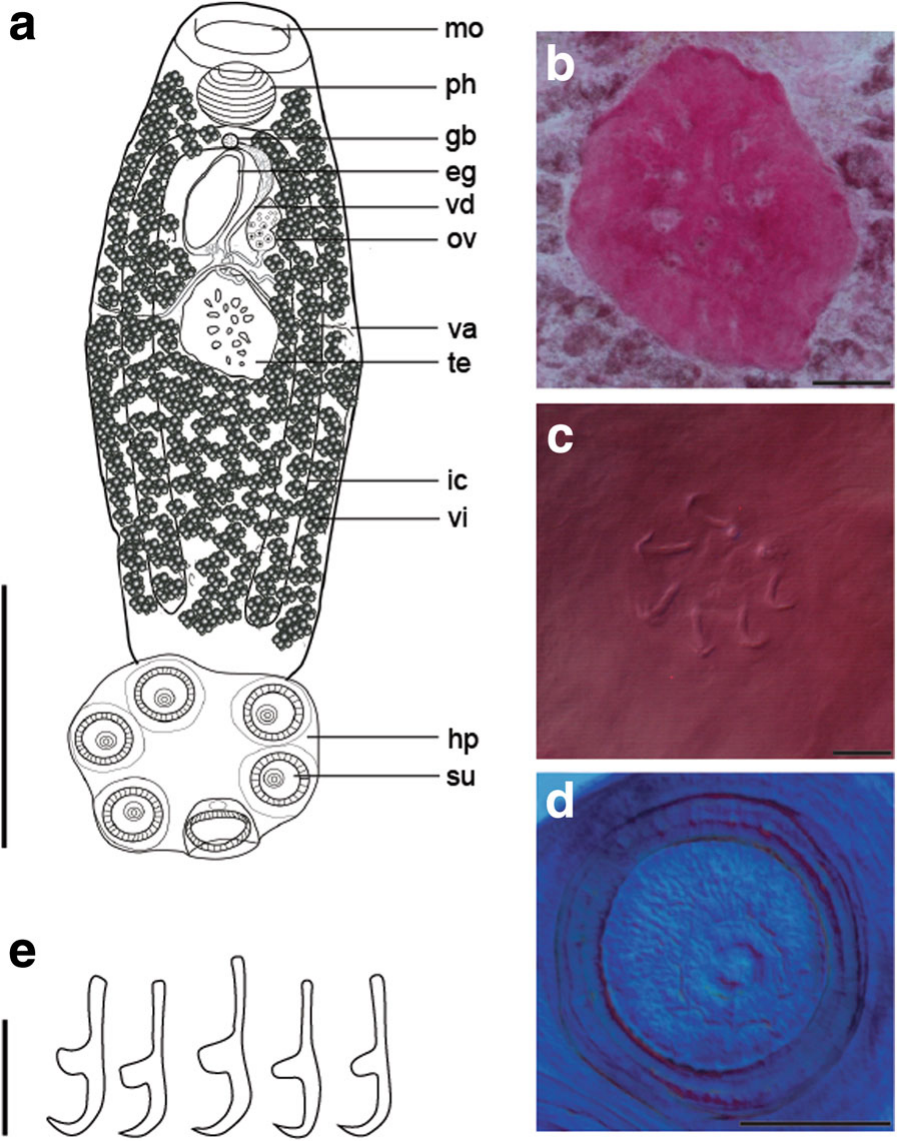
*Neopolystoma guianensis* n. sp. Holotype. **a**, Ventral view. **b**, testis. **c** genital spines. **d** haptoral sucker showing a ring of skeletal elements. **e** marginal hooklets. *Abbreviations:* eg, egg; gb, genital bulb; hp, haptor; ic, intestinal caecum; mo, mouth; ov, ovary; ph, pharynx; su, sucker; te, testis; va, vagina; vd, vas deferens; vi, vitellaria. *Scale-bars*: **a**, 1,000 μm; **b**, 100 μm; **c**, 10 μm; **d**, 100 μm; **e**, 10 μm

[Based on 11 egg-producing adults (see Table 1 and Fig. 2a-e). No larval measurements or characters are given as eggs collected from the host failed to develop.] Body elongate, spindle shaped, with widest point in middle of body proper, total length 2,284–3,342 (2,737), greatest width 830–1,164 (1,037). Haptor length 634–852 (755), haptor width 784–1014 (910), haptor length to body length ratio 0.24–0.31 (0.28). Mouth subterminal, ventral. False oral sucker prominent, 189–391 (290) wide. Pharynx 189–260 × 226–283 (213 × 252). Intestine bifurcates without diverticula; caeca narrow, lacking anastomoses, run laterally, not confluent posteriorly, not extending into haptor. Testis single, spherical, compact (Fig. 2b), mid-ventral, medial, posterior to ovary (Fig. 2a), 364–541 × 380–527 (437 × 477). Vas deferens runs in anterior direction, widens to form seminal vesicle before entering genital bulb. Genital atrium median, ventral, posterior to intestinal bifurcation, 49 in length, with 8 spines, 8.4–9.8 (8.9) long (Fig. 2c); spines slightly curved. Ovary about 1/3 from the anterior extremity, dextral, small, 149–243 × 71–120 (197 × 97). Uterus short, tubular, anterior to ovary, contained one fusiform egg *in utero* (all specimens studied); egg capsule 280–344 × 127–163 (317 × 148). No intrauterine development observed, eggs operculate. Vaginae present, located nearly in middle of body proper; body width at the level of vagina 792–1,091 (989). Vitellarium extends from just behind pharynx throughout most of body proper except for area around gonads and posteriormost part of body proper. Genito-intestinal canal obscured by testis, dextral, joining intestinal caecum posterior to ovary (Fig. 2a). Live specimens were extremely flexible and able to extend the body proper to nearly double its length. Posterior section of body proper appears to be especially very flexible and elastic. Haptoral suckers 6, muscular, with well-developed skeletal structure inside (Fig. 2d), mean diameter 208–242 (226). Hamuli absent. Marginal hooklets retained in adult parasites (Fig. 2e). Posteriormost marginal hooklet 1 13.3–15.1 (14.3) long; hooklets 2–8 12.9–14.3 (13.9) long.

### *Neopolystoma scorpioides* n. sp.

***Type-host*** **:** *Kinosternon scorpioides* (Linnaeus, 1766) (Kinosternidae Agassiz, 1857).

***Type-locality*** **:** Forest pond along the Cayenne-Kaw road south of the town of Roura, French Guiana (4.66997N, 52.30560W).

***Site in host*** **:** Conjunctival sacs of the eye.

***Type-material***: Three sexually mature worms. The holotype (NMB P407) and two paratypes (NMB P408–P409) were deposited in the Parasitic Worm Collection, National Museum, Aliwal Street, Bloemfontein, South Africa.

***Prevalence and intensity*** **:** Site D: Prevalence 7.7%; One specimen infected by four polystomes.

***Representative DNA sequences*** **:** GenBank accession numbers: KY200990 (28S), KY200993 (12S) and KY200996 (*cox*1).

***ZooBank registration***: To comply with the regulations set out in article 8.5 of the amended 2012 version of the *International Code of Zoological Nomenclature* (ICZN) [33], details of the new species have been submitted to ZooBank. The Life Science Identifier (LSID) of the article is urn:lsid:zoobank.org:pub:B55369A7-5 F88-4A72-94 F2-E0CE354484A1. The LSID for the new name *N. scorpioides* n. sp. is urn:lsid:zoobank.org:act:7852C6EA-FDEC-435 F-9319-5379747 F7099.

***Etymology*** **:** This parasite is named after the host *K. scorpioides*.

### Description

**Fig. 3:**
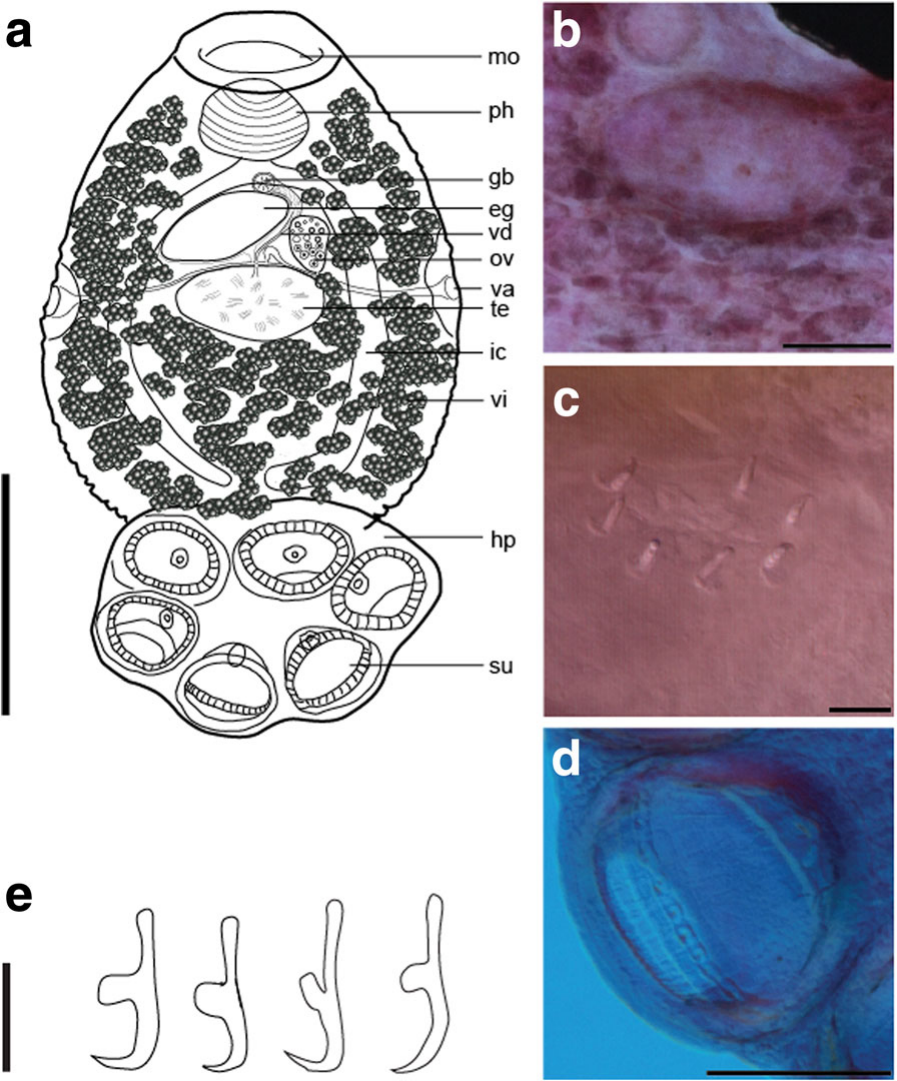
*Neopolystoma scorpioides* n. sp. Holotype. **a** Ventral view. **b** testis of holotype. **c** genital spines. **d** haptoral sucker showing a ring of skeletal elements. **e** marginal hooklets. *Abbreviations*: eg, egg; gb, genital bulb; hp, haptor; ic, intestinal caecum; mo, mouth; ov, ovary; ph, pharynx; su, sucker; te, testis; va, vagina; vd, vas deferens; vi, vitellaria. *Scale-bars*: **a**, 500 μm; **b**, 100 μm; **c**, 10 μm; **d**, 100 μm; **e**, 10 μm

[Based on three egg-producing adults (see Table 1 and Fig. 3a-e). No larval measurements or characters are given as eggs collected from the host failed to develop.] Body oval, total length 1,512–1,770 (1,658), greatest width 868–907 (894), width at vagina 837–876 (863). Haptor length 445–511 (485), haptor width 719–765 (735), haptor length to body length ratio 0.29. Mouth subterminal, ventral. False oral sucker prominent, 240–245 (242) wide. Pharynx 170–184 × 226–231 (177 × 228). Intestine bifurcates without diverticula; caeca lacking anastomoses, not confluent posteriorly, not extending into haptor. Testis single, compact, oval (Fig. 3b), mid-ventral, medial and posterior to ovary, 120–142 × 246–301 (127 × 271). Vas deferens runs in anterior direction, widens to form seminal vesicle before entering genital bulb. Genital atrium median, ventral, posterior to intestinal bifurcation, 45 in diameter, with eight straight spines with curved tips (Fig. 3c), 5.5–8.7 (7.7) long. Ovary dextral, at 44% of body length from anterior extremity, 136–187 × 66–71 (153 × 69). Uterus short, tubular, anterior to ovary, containing only one fusiform egg; egg capsule 253–279 × 133–156 (265 × 143). No intrauterine development, eggs operculate. Vitellarium extends from just behind pharynx throughout most of body proper except for area around gonads and posteriormost part of body proper. Genito-intestinal canal obscured by testis, dextral, joining intestinal caecum posterior to ovary (Fig. 3a). Haptoral suckers 6, muscular, with well-developed skeletal structure inside (Fig. 3d), mean diameter 133–279 (220). Hamuli absent. Marginal hooklets (Fig. 3e) retained in adult parasites. Posteriormost marginal hooklet 1 16.4–17.2 (16.8) long; hooklets 2–8 13.4–14.8 (14.2) long.

### Molecular, phylogenetic and genetic divergence analyses

**Fig. 4:**
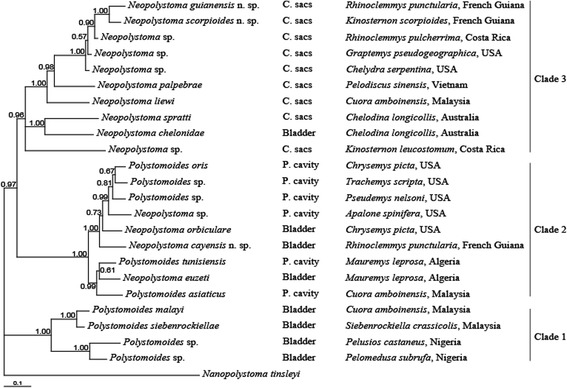
Bayesian tree inferred from the analysis of four concatenated genes. Numbers at nodes correspond to Bayesian posterior probabilities. *Abbreviations*: C. sacs, conjunctival sacs; P. cavity, pharyngeal cavity

One worm of each of *N. cayensis* n. sp., *N. guianensis* n. sp. and *N. scorpioides* n. sp. were used for molecular studies. With the exception of the 18S rRNA and a portion of the 28S rRNA genes that failed to amplify for *N. scorpioides* n. sp., we obtained sequences for all three polystomes. The Bayesian tree showed with high confidence values the phylogenetic relationships within chelonian polystomes (Fig. 4). *N. guianensis* n. sp. and *N. scorpioides* n. sp. were sister species and nested in Clade 3, according to Héritier et al. [21]. Estimates of *cox*1 genetic divergences between these two polystomes were about 15.5%, which was well beyond the threshold of 3.4% defined for chelonian polystomes by Héritier et al. [23]. These two polystomes thus belonged to separate species. Conversely, *N. cayensis* n. sp. was nested in Clade 2 according to Héritier et al. [21]. Estimates of *cox*1 genetic divergences between this polystome and the former two species were 22.2 and 26.1%, respectively, and ranged from 16.9 to 20.3% with the most closely related species. These results also indicated that this polystome belonged to another species.

## Discussion

### Diagnosis

Whereas two *Neopolystoma* species were known from Central America and so in the Neotropical realm, namely *Neopolystoma fentoni* Platt, 2000 from Costa Rica and *Neopolystoma domitilae* (Caballero, 1938) from Mexico, the three *Neopolystoma* species described herein are the first members of the genus recorded from South America. *Neopolystoma cayensis* n. sp. differs from other members of the genus reported in the Neotropical realm by a combination of characters. Based on the body length, *N. cayensis* n. sp., with a length of 3,342–5,575 (4,139) μm, is significantly longer than *N. guianensis* n. sp. with a length of 2,284–3,342 (2,737) μm, *N. scorpioides* n. sp. with a length of 1,512–1,770 (1,658) μm and *N. fentoni* with a length of 1,500–2,450 (1,985) μm*.* If it is not significantly longer than *N. domitilae* (4,039–4,057 μm), the latter is significantly longer than *N. guianensis* n. sp. and *N. scorpioides* n. sp. Based on the genital spine number, *N. cayensis* n. sp. with 16–17 spines differs from *N. domitilae* with 20–21 spines and from the three polystomes of the conjunctival sacs, namely *N. guianensis* n. sp., *N. scorpioides* n. sp. and *N. fentoni*, which all have eight spines. Based on the genital spine length, *N. cayensis* n. sp. with spine length of 19–26 (23) μm also differs from *N. domitilae* with spine length of 55–71 μm, *N. fentoni* with spine length of 11 μm, *N. guianensis* n. sp. with spine length of 8.4–9.8 (8.9) μm and *N. scorpioides* n. sp. with spine length of 5.5–8.7 (7.7) μm. Concerning testis length, it varies between all *Neopolystoma* species. *Neopolystoma cayensis* n. sp. has a testis length of 560–983 (762) μm, *N. guianensis* n. sp. a testis length of 364–541 (437) μm, *N. domitilae* a testis length of 450–664 μm, *N. fentoni* a testis length of 98–367 (225) μm and *N. scorpioides* n. sp. a testis length of 120–142 (127) μm.

### Diversity of chelonian polystomes in South America

In spite of the considerable diversity of South American amphibians [34] and chelonians [4], relatively few polystomes were reported from South America [20, 22]. Currently, 16 polystomes are known from anurans, three from caecilians and six from chelonians. Turtle polystomes include *Polystomoides coronatum* (Leydi, 1888) from *Trachemys dorbigni* (Duméril & Bibron, 1835); *Polystomoides uruguayensis* Mané-Garzon & Gil, 1961 from *Phrynops hilarii* (Duméril & Bibron, 1835); *Polystomoides fuquesi* Mané-Garzon & Gil, 1962 from *P. hilarii*; *Polystomoides rohdei* Mané-Garzon & Holcman-Spector, 1968 from *T. dorbigni*; *Polystomoides brasiliensis* Vieira, Novelli, Souza & de SouzaLima, 2008 from *Hydromedusa maximiliani* (Mikan, 1820) and *Phrynops geoffroanus* (Schweigger, 1812); and *Polystomoides magdalenensis* Lenis & Garcia-Prieto, 2009 from *Trachemys callirostris* (Gray, 1856). All of these were reported from the pharyngeal cavity of their host. If we assume, as stated by Verneau [22], that chelonian polystomes are host- and site-specific in natural environments on the one hand and that turtle species are infected by no more than three polystome species depending of their ecological niche on the other, it is likely that a single polystome species rather than two really infect each of the two freshwater turtles *T. dorbigni* and *P. hilarii*, respectively. We should therefore consider only four *Polystomoides* species occurring within South American chelonian hosts. However, this must be further confirmed from genetic analyses of the *cox*1 marker. The description of three new species in this study, namely *N. cayensis* n. sp., *N. guianensis* n. sp. and *N. scorpioides* n. sp. is a novelty as it is the first statement of polystomes from the bladder and conjunctival sacs of South American turtles. Although Avila et al. [35] reported the presence of a *Neopolystoma* species from *Mesoclemmys vanderhaegei* (Bour, 1973) in Brazil, nothing was mentioned about the ecological niche of this undescribed parasite. We can, therefore, consider that *Neopolystoma* is here accounted for the first time in South America. Knowing that we surveyed in just two weeks 68 specimens of freshwater turtles belonging to three distinct species and in the light of the rich diversity of chelonians in South America [4], it is very likely that vast numbers of polystomes remain undescribed.

### Status of *Neopolystoma* and *Polystomoides*

Whereas the three chelonian polystome genera *Neopolystoma, Polystomoides* and *Polystomoidella* are distinguished on the basis of the presence of hamuli, i.e. *Neopolystoma* has no hamuli*, Polystomoidella* has a single pair of hamuli and *Polystomoides* has two pairs, it is now well established that *Neopolystoma* and *Polystomoides*, at least, are not monophyletic [21, 36]. The phylogenetic study by Héritier et al. [21] showed that the absence of hamuli on the one hand and the presence of two pairs of hamuli on the other were not true morphological synapomorphies for *Neopolystoma* and *Polystomoides*, respectively. Whereas *Neopolystoma* and *Polystomoides* are known from the conjunctival sacs, bladder and pharyngeal cavity of their host [22], *Polystomoidella* is known only from the bladder of its host. Regarding our phylogenetic tree, all *Neopolystoma* spp. from the conjunctival sacs could form a distinct clade, i.e. Clade 3 (Fig. 4), if the possibility is considered of an accidental switch of vials or a mislabelled tissue when sampling *Neopolystoma chelodinae* (MacCallum, 1918), which infects the bladder of *Chelodina longicollis* (Shaw, 1794) from Australia and which is nested within that clade. Based on morphology, *Neopolystoma* spp. from the conjunctival sacs do not differ significantly from any other chelonian polystomes from the bladder and pharyngeal cavity. However, parasites of the conjunctival sacs are the only chelonian polystomes that do not lay spherical eggs. They indeed produce fusiform or spindle-shaped eggs [24]. We can therefore hypothesise that this particular shape of egg could be a synapomorphy for the group of polystomes infecting the conjunctival sacs. However, this needs to be further validated after a more in-depth study of polystomes collected from Australian turtles, particularly *N. chelodinae* from *C. longicollis*. Clade 1 of the Bayesian tree contains only polystomes with two pairs of hamuli, i.e. *Polystomoides* spp. that infect the bladder of their host. Although species of *Polystomoides* can be also found within Clade 2, all of them infect the pharyngeal cavity of their host. Hence, this suggests that polystomes with two pairs of hamuli occurring in the bladder of their host belong to a separate lineage (Clade 1). On the opposite, Clade 2 of the Bayesian tree contains species of both genera, with *Polystomoides* spp. infecting the pharyngeal cavity and *Neopolystoma* spp. infecting either the pharyngeal cavity or the bladder. Because there is no trend in the number of hamuli and the ecological niche of the parasites nested in Clade 2, the morphology of these species should be revisited in order to establish whether morphological synapomorphies for that particular clade really exist.

### The need for the study of helminth diversity

In the pursuit of conservation efforts in any vertebrate group, it is important to understand the broad biological context of that group, including its parasite diversity. While some parasites are harmless to chelonian hosts, others may adversely affect host behaviour, general fitness and chances of survival [13, 14]. Furthermore, in a changing world where animals are sold for pets, some species may invade new environments and bring with them a great number of parasite species. Concerning turtles, Meyer et al. [25] and Héritier et al. [23] investigated the polystome richness among two European freshwater turtles, *Mauremys leprosa* (Schweigger, 1812) and *Emys orbicularis* (Linnaeus, 1758), respectively. They documented a greater polystome diversity than expected assuming a high degree of host- and site-specificity [22]. Surprisingly, a few polystome species reported within populations of *M. leprosa* and *E. orbicularis* were also found in American hosts that had never been recorded in the European freshwater environments of investigation. Meyer et al. [25] and Héritier et al. [23] showed that these parasite species were in fact introduced into natural wetlands following the introduction of the American red-eared slider *T. s. elegans*. Because that turtle is among the most common exotic turtle species in captivity, it would act as a reservoir of numerous parasite species [24], and, once released into freshwater habitats, it would have served as a carrier of parasites, as for instance *Neopolystoma* sp. 4 (haplotype H18), *Polystomoides* sp. 1 (haplotype H16) and X. sp (haplotype H36), which would have been transmitted to native turtles across European freshwater environments [23, 25]. Since *T. s. elegans* was banned from the commercial trade in Europe, some other turtle species were identified in pet markets. Among the 17 chelonian species that were offered for sale by the French society “La Ferme Tropicale” on July 2016 07th (https://www.lafermetropicale.com), 13 freshwater species were from the USA, Central America, Asia and Africa. A similar situation was found on the online Reptimania society (http://www.reptimania.com). On July 2016 07th, numerous exotic chelonian species were indeed offered for sale, among which *R. pulcherrima*. Hence, this demonstrates the need to document and describe as fast as possible the helminth diversity of freshwater turtles in their home range before their parasites invade novel freshwater environments following the introduction and possible release of new exotic chelonian species.

## Conclusions

Based on morphological and molecular characters, three new polystome species are described therein from freshwater turtles of French Guiana, i.e., *K. scorpioides* and *R. punctularia*. These are the first representatives of Neopolystoma in South America. Nevertheless, regarding our global phylogenetic analysis of chelonian polystomes, the revision of *Polystomoides* and *Neopolystoma* taxonomy within the Polystomatidae is required. In fine, as more and more South American turtle species are considered in the international pet trade, our study indicates the importance to survey parasite diversity within chelonians in their home range, before they are released in new freshwater environments.
